# The Relationship Between Spirituality, Health-Related Behavior, and Psychological Well-Being

**DOI:** 10.3389/fpsyg.2020.01997

**Published:** 2020-08-14

**Authors:** Agnieszka Bożek, Paweł F. Nowak, Mateusz Blukacz

**Affiliations:** ^1^ Institute of Psychology, Jagiellonian University, Cracow, Poland; ^2^ Faculty of Physical Education and Physiotherapy, Opole University of Technology, Opole, Poland; ^3^ Institute of Psychology, University of Silesia in Katowice, Katowice, Poland

**Keywords:** spirituality, health-related behavior, psychological well-being, acquired education, public health

## Abstract

Studies suggest a positive association of spirituality and health behaviors with well-being (especially subjective well-being), but still the precise character of such relationships between all these constructs remains unknown. The present study aims to explore the relations between spirituality, health-related behaviors, and psychological well-being in the context of acquired education. A questionnaire survey was conducted among 595 students from six different universities, whose study programs either focused on the human body or the human mind and spirit. Path analysis and linear regression were used to model the relationship between the examined constructs. The results show that both spirituality and health-related behaviors are positively related to psychological well-being, and that the relationship with spirituality is also mediated by health-related behaviors. Only spirituality is associated with the type of acquired education, especially in the group of students whose studies focus on the human mind and spirit. Moreover, spirituality in this group seems to display a stronger relationship with psychological well-being. These findings may contribute to the better understanding of some significant determinants of psychological well-being. They carry important implications for the faculty members responsible for curriculum preparation to account for teaching contents related to the conduct of a healthy lifestyle and to spiritual development.

## Introduction

Almost 20 years ago, following the commencement of the positive psychology movement, the research approach in the areas of psychology, especially those concerning mental health, began to change, concentrating on a much greater interest in well-being than on mere diseases or disorders (Bhullar et al., 2014). Two main directions have emerged in well-being research: one based on a hedonistic approach and the other on eudaimonia. According to the hedonistic approach, well-being is concerned with affective pleasure in someone’s life (Watson et al., 1988). The term subjective well-being (SWB) is used in positive psychology in the sense of a high level of positive affect, a low level of negative affect, and a high degree of satisfaction with one’s life (Deci and Ryan, 2008). In contrast, in the eudaemonist approach, well-being is perceived as the degree to which people function so that they could realize their full potential (Waterman, 1993). In publications on positive psychology eudaimonism is often synonymous with psychological well-being (PWB; Ryan and Deci, 2001).

Recently, the main direction in studies of determinants of well-being has focused on subjective well-being (Diener, 2000). Demographic determinants (Argyle, 1999), cognitive and motivational determinants (Lyubomirsky, 2001), and personality determinants (Park, et al., 2004) of subjective well-being have been identified. Less attention is currently paid to psychological well-being, however, some research indicated religion and spirituality to be significant PWB implications (Levin and Chatters, 1998; Lawler-Row and Elliott, 2009) and revealed associations between pro-health behaviors, spirituality, and well-being (Boswell et al., 2006). At present, in a holistic view of health beyond biological and psychosocial well-being, the spiritual dimension of well-being is frequently discussed. This new construct is defined as a sense of connection with others, sense of life, and relationship with a transcendent force. It has psychosocial and religious components, and it is believed to promote spiritual health (Ghaderi et al., 2018; Alborzi et al., 2019). The present study attempts to gain a better insight into relationships between spirituality, health-related behavior, and psychological well-being with regard to the type of acquired education.

## Theoretical Background

### Psychological Well-Being

The answer to the question “What does it mean to feel well psychologically?” needs to be sought in literature on humanistic psychology, including developmental and health psychology (Ryff, 1989). Ryff created a multidimensional construct of well-being, building on such concepts as basic life tendencies of Buhler (1935), psychosocial stages of Erikson (1959), personality changes of Neugarten (1973), positive criteria of mental health of Jahoda (1958), account of individuation of Jung (1933), formulation of maturity of Allport (1961), depiction of the fully-functioning person of Rogers (1961), and notion of self-actualization of Maslow (1968).

Psychological well-being covers a wide range of welfare including positive assessments of oneself and one’s past life (Self-Acceptance), a sense of continued growth and development as a person (Personal Growth), the belief that one’s life is purposeful and meaningful (Purpose in Life), the possession of quality relations with others (Positive Relations With Others), the capacity to manage effectively one’s life and the surrounding world (Environmental Mastery), and a sense of self-determination (Autonomy; Ryff and Keyes, 1995, p. 720). Ryff and Singer (1998) also developed a measure to assess the above six distinct factors of positive psychological functioning.

Both the model and the measure came under review. The former was criticized for the lack of independence of individual scales (Springer and Hausner, 2006). According to various researchers, Personal Growth, Purpose in Life, Self-Acceptance, and Environmental Mastery do form a single scale. The latter was criticized for its lack of factorial validity or internal consistency (van Dierendonck, 2004). However, some other studies supported the six-factor PWB model (Ryff and Singer, 2006; van Dierendonck et al., 2008) and also revealed the existence of a single higher-order PWB factor above the subscales (Keyes et al., 2002).

The concept of PWB corresponds to the WHO definition of health as a state of complete physical, mental, and social well-being, not merely the absence of disease or infirmity, formulated in 1948 (WHO, 1948). A high level of PWB is associated with a lower risk of depression (Ryff and Keyes, 1995; Fava, 1999), a lower possibility of displaying risk behavior (Yonker et al., 2012), and a decreased immune cell expression of a conserved transcriptional response to adversity (CTRA; Fredrickson et al., 2015).

### Spirituality

According to Joseph et al. (2017, p. 506), spirituality should be understood as “a more general, unstructured, personalized, and naturally occurring phenomenon, where a person seeks closeness and/or connectedness between him/herself and a higher power or purpose.” Other authors define spirituality in terms of search for universal truth and as an activity enabling people to discover meaning and significance in the surrounding world (Woods and Ironson, 1999). Spirituality can also be perceived as a dynamic reality, constantly exploring something new; it may also involve the learning of the ultimate boundaries of existence and seeking a broader meaning of life. Hart (1994, p. 23) defined spirituality as a way in which an individual experiences his or her faith in everyday life and style “in which the individual refers to the final conditions of individual existence.”

Spirituality therefore forms a multidimensional theoretical construct. In essence, it constitutes transcendence understood as going beyond or above “the real I.” In this context, spirituality is defined as experiencing transcendence through inner peace, harmony, or connectedness to others (Boswell et al., 2006). Transcendence can take place both within the person (self-realization, self-improvement, and personal development) and outside the person. “External” transcendence may be directed to a higher entity or energy; to another person, claimed to be of particular value, whose good is more important than one’s own good; or to the universe (Heszen-Niejodek and Gruszyńska, 2004). Spirituality differs from religion as the latter is rather linked with specific rituals, institutional dependencies, and social relationships, whereas the former is more about personal experience of what is unseen and recognized as greater than ourselves (Tovar-Murray, 2011). Thoresen (1998) claims that religion is perceived mainly as a social phenomenon, while spirituality is usually considered at the individual level and within a specific context. Despite their common transcendence-related roots, spirituality and religiosity may not be treated interchangeably. These are different areas, however, overlapping in their meaning (Krok, 2009a).

Heszen-Niejodek and Gruszyńska (2004) understand transcendence as a common denominator for many concepts of spirituality. The two-way understanding of transcendence, described above as self-improvement and as a turn toward a higher-being, makes it possible to examine the phenomenon of spirituality using the methodology of psychological sciences, without questioning theological and philosophical perspectives (Krok, 2009a).

Studies demonstrated the positive impact of spirituality on physical health and mental health as well as on other positive health outcomes such as subjective well-being, health-related quality of life, coping skills, recovering from mental illness, or less addictive or suicidal behaviors (Mueller et al., 2001; Miller and Thoresen, 2003; Kharitonov, 2012; Unterrainer et al., 2014). However, we must bear in mind that spirituality is a complex construct and as such it is defined in multiple ways and measured with different tools (Lun and Bond, 2013).

In our study, we used the Self-Reported Questionnaire by Heszen-Niejodek and Gruszyńska (2004), in which the overall factor Spirituality consists of Religious Attitudes (religious experiences, their importance in everyday life, their influence on moral choices and behavior, and relationship to God); Ethical Sensitivity (high place of ethical values in the hierarchy of values, our compliance with them, and tendency toward ethical reflection); and Harmony (seeking harmony with the world, internal consistency, and cohesion of various forms of one’s own activity). These dimensions reflect the main manifestations of spirituality available in internal experience, distinguished on the basis of descriptions of specific manifestations of spirituality in psychological literature (Hill et al., 2000; Socha, 2000; Thoresen and Harris, 2002), subsequently ordered according to the aforementioned directions of transcendence (me, God, other people, and the world).

### Health-Related Behavior

Lifestyle and lifestyle-related health behaviors are some of the determinants of health potential (Binkowska-Bury et al., 2010). A health behavior is any activity undertaken to prevent or detect disease or to improve health and well-being (Conner and Norman, 1996). In studies on health behavior and behavioral change, health behaviors are usually divided into those associated with physical activity, diet, and the use of psychoactive substances (Norman et al., 2008). However, there are currently other more popular approaches that consider multiple lifestyle-forming health behaviors, between which different interactions take place (op. cit).

The present study uses an approach that distinguishes four categories of health-related behaviors: (a) proper nutrition habits (eating proper food and keeping a well-balanced diet); (b) prophylaxis (obeying health recommendations and obtaining health and disease information); (c) positive attitude (avoiding emotional overload, stress, or depressing situations); and (d) pro-health practices (good sleeping habits, relaxation, and physical activity; Juczyński, 2009).

The positive impact of health-related behavior on subjective well-being has already been the subject of studies on various age groups: adolescents (Shaffer-Hudkins, 2011; Sacker, 2012), university students (Binkowska-Bury et al., 2010), and older adults (Boswell et al., 2006). However, little is still known about the precise relationship between health-related behavior and psychological well-being. Moreover, there have been very few studies regarding the impact of spirituality on health-related behavior, although the former was recognized as one of four dimensions of health (Harris et al., 1999).

## Present Study

**Figure 1:**
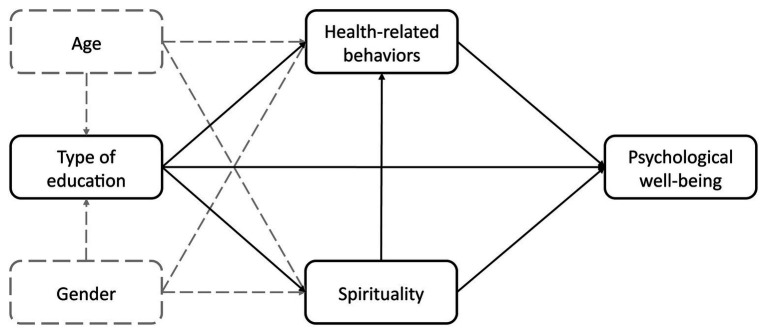
The structure of the theoretical model. Solid lines designate theoretical relations of interest and dashed lines represent control variables.

The main aim of this study was to examine the relationship between spirituality, health-related behavior, type of acquired education, and psychological well-being. To achieve this goal, a path analysis was conducted. It is considered one of a few possible statistical approaches addressing the problem of spirituality and health, recommended by Miller and Thoresen (2003). The path model helps to verify assumed relationships between a set of variables represented as a structure of the tested model, which is based on theoretical associations between the variables. It also provides a framework for the analysis of the direct, indirect, and total effects, whose form provides an analytical basis for interpreting moderation effects (Alwin and Hausner, 1975; Miller and Thoresen, 2003). Direct effects are regression coefficients representing the structural components of the model; indirect effects are parts of causal influence transmitted by intervening moderator and mediator variables; and total effects are the totals of direct and indirect effects (Alwin and Hausner, 1975; Pearl, 2012). It must be highlighted that although path analysis implies causality, it cannot be inferred from the gathered data since it is cross-sectional (Bollen and Pearl, 2013). The directions of the relations in the model were based on the mentioned literature, yet the model itself was not aimed at testing causal effects. The path analysis was used to disaggregate, quantify, and compare the magnitude of associations between the variables (Miller and Thoresen, 2003; Bollen and Pearl, 2013). The model used in this study is illustrated in Figure 1.

The focus of this model was to estimate a hypothetical structure of associations of psychological well-being, health-related behaviors, spirituality, and type of acquired education, with age and sex being two control variables.

### General Hypothesis

On the basis of existing research (Levin and Chatters, 1998; Boswell et al., 2006; Lawler-Row and Elliott, 2009; Yonker et al., 2012; Archana and Updesh, 2014), we assumed that spirituality and health-related behaviors were factors which could be positively associated with psychological well-being. We also supposed that both factors had a positive relationship with subjective well-being. Moreover, since spirituality has been proven to reduce the odds of health-risk behavior (Jesse and Reed, 2004; see also Unterrainer et al., 2014) and is associated with a higher level of health-behavior (Park et al., 2009); we hypothesized that spirituality would also be associated with health-related behavior.

Researchers indicate that the acquired knowledge about health has a significant impact on exhibited health behaviors (White et al., 2009; Muennig et al., 2011; Sørensen et al., 2012; Yokokawa et al., 2016). Thus, the university students who were invited to take part in our research attended study programs primarily concerned with either physical health and the human body or with psychosocial health and the human mind and spirit. The first group comprised students whose study curricula included primarily biological sciences subjects such as anatomy, human physiology, biomechanics as well as other professional courses preparing students to pursue a professional career in, for example, kinesiology. The second group consisted of students whose study curricula included subjects in the humanities and social sciences such as developmental psychology, social psychology, psychology of mental disorders, philosophy, etc. In the first group, the acquired knowledge predisposed students to develop a biomedical approach to health; in the second group, the emphasis was placed on psychosocial health and, consequently, on developing a socio-ecological approach to health.

It could be assumed that studies that prepare for physical health-related occupations may contribute to a large extent to the development of a body-centric approach in the students. Among many possible health behaviors, there are those directly related to the biological dimension of health, including physical activity and diet, and less concentrated on psychosocial skills and behaviors. Conversely, students educated in the humanities and social sciences, due to their dominant curriculum subjects, may be more focused on developing their potential in the area of psychosocial and spiritual health rather than physical health.

## Materials and Methods

### Participants

The study was conducted among 595 students from six Polish universities: 295 majoring in physical health, physiotherapy, and tourism and recreation (education about the human body) and 300 students majoring in psychology, pedagogy, or theology (education about the human mind and spirit). The study comprised 387 (65%) women and 208 (35%) men, aged 18–30 years (*M* = 21.67; *SD* = 1.88). Table 1 contains descriptive statistics of the variables used in the study.

**Table 1:** Mean and standard deviations for the whole group and split groups according to students’ type of education and gender.

	Entire group (*n* = 595)				Human body education (*n* = 295)					Mind and spirit education (*n* = 300)					Female students (*n* = 387)					Male students (*n* = 208)				
Variable	M	SD	Min	Max	M	SD		Min	Max	M	SD	Min		Max	M	SD	Min		Max	M	SD	Min	Max	
Age	21.73	1.99	18.00	30.00	21.15	1.56		18.00	29.00	22.30	2.20	18.00		30.00	21.70	1.72	18.00		30.00	21.78	2.42	18.00	30.00	
**Health-related behaviors**																				
Sum score	77.77	12.52	30.00	120.00	77.18	12.11	30.00		103.00		78.35	12.90	46.00	120.00		79.25	12.53	30.00	120.00	75.02	12.05	43.00		109.00	Prop. nutr. habits	3.20	0.82	1.17	5.00	3.11	0.75	1.33	5.00	3.29	0.87	1.17	5.00	3.34	0.80	1.33	5.00	5.00	0.80	1.17		5.00	Prophylaxis	3.04	0.74	1.00	5.00	3.03	0.76	1.00	4.50		3.05	0.73	1.33	5.00	3.09		0.72	1.00	5.00	2.96	0.78	1.17		4.50	Positiveattitudes	3.43	0.66	1.17	5.00	3.45	0.63	1.50	5.00		3.42	0.69	1.17	5.00	3.45		0.66	1.50	5.00	3.40	0.66	1.17	5.00	Pro-healthpract.	3.28	0.66	1.17	5.00	3.27	0.63	1.17	4.67	3.30		0.68	1.50	5.00	3.33	0.67	1.17	5.00	3.20	0.62	1.17		4.50
**Spirituality**																					Spirituality sum	3.57	0.58	1.86	5.00	3.41	0.55	1.86	4.75	3.73	0.56	1.86	5.00	3.60	0.54	1.86	5.00	3.52		0.65	1.86	4.94	Relig. Attitudes	3.52	0.73	1.14	5.00	3.32	0.70	1.14	4.86	3.72	0.70	1.29	5.00	3.57	0.65	1.29	5.00	3.42	
0.84				1.14	5.00	Ethic. Sensitivity	3.76	0.55	1.71	5.00	3.63	0.54	2.00	5.00	3.89	0.53	1.71	5.00	3.79	0.51	2.14	5.00	3.72	0.62	1.71		5.00	Harmony	3.44	0.68	1.00	5.00	3.29	0.65	1.00	4.83	3.59	0.68	1.50	5.00	3.45	0.68	1.00	5.00	3.42	0.68	1.83		5.00
**Psychologicalwell-being**																					Sum score	4.53	0.32	3.50	5.50	4.52	0.31	3.52	5.50	4.55	0.33	3.50	5.40	4.53	0.32	3.50	5.40	4.53	0.32	3.52	5.50		Authonomy	4.73	0.92	1.71	7.00	4.77	0.92	1.71	7.00	4.70	0.92	2.00	6.86	4.62	0.90	1.71	6.86	4.94	0.92	2.57	7.00		Environ. Mastery	4.81	0.91	1.86	7.00	4.81	0.86	2.14	7.00	4.81	0.97	1.86	7.00	4.79	0.90	1.86	7.00	4.85	0.95	1.86	7.00		Personal Growth	4.95	0.76	2.43	6.86	4.81	0.74	2.86	6.86	5.08	0.77	2.43	6.86	4.96	0.75	2.43	6.86	4.93	0.80	2.86	6.86		Pos. Relations with Others	5.42	0.89	2.00	7.00	5.33	0.86	2.00	7.00	5.51	0.90	2.57	7.00	5.51	0.89	2.00	7.00	5.24	0.87	2.57	7.00		Purpose in Life	5.00	0.91	2.29	7.00	4.82	0.92	2.29	7.00	5.18	0.87	2.43	7.00	5.06	0.87	2.86	7.00	4.89	0.97	2.29	7.00		Self-Acceptance	4.76	0.98	1.43	7.00	4.71	0.93	1.43	7.00	4.80	1.03	1.43	7.00	4.73	0.98	1.43	7.00	4.80	0.98	2.29	7.00	

The research was carried out at selected universities in southern and central Poland. After obtaining the consent of the management of a given university institute and the lecturer in charge of the class, on a designated day, the researchers asked students to fill in a set of questionnaires. Each study lasted 30 min on average. After completing the questionnaires the collected data were transferred to a spreadsheet and double-checked.

### Measures

#### Psychological Well-Being

The PWB measure is based on the eudemonistic concept of well-being developed by Ryff (1989). In our study, we used Polish adaptation by Krok (2009b). The questionnaire contains 42 items in six subscales: Self-Acceptance, Personal Growth, Purpose in Life, Positive Relations with Others, Environmental Mastery, and Autonomy. The items are assessed on a 7-point Likert scale (from 1 – strongly disagree to 7 – strongly agree). It is also possible to calculate the general factor of psychological well-being as a mean value of six subscales. The internal consistency indicator for the whole scale was *α* = 0.914.

#### Self-Report Questionnaire

The self-report questionnaire was developed by Heszen-Niejodek and Gruszczyńska (2004) and Metlak (2002) to measure the level of spirituality. It consists of 20 statements assessed on a 5-point Likert scale (from 1 – definitely not to 5 – definitely yes). The results are calculated separately for the whole scale as well as for three individual subscales: Religious Attitudes (sample item: “I feel God’s love for me directly or through other people”), Ethical Sensitivity (sample item: “When making decisions, I wonder if I’m acting morally”), and Harmony (sample item: “I feel deep inner peace”). The reliability indicator for the spirituality scale was *α* = 0.903.

#### Inventory of Health-Related Behavior

This questionnaire is intended to measure health behaviors and contains five scales: a general health behaviors rate and its four indicators: proper nutrition habits, prophylaxis, positive attitude, and pro-health practices. The inventory was developed by Juczyński (2009) based on terms of health behaviors developed by Gochman (1988) and available tools for testing health practices including the Reported Health Behaviors Checklist (Prohaska et al., 1985). It contains 24 statements describing various types of health-related behaviors (sample items: “I avoid consuming food with preservatives,” “I regularly apply for medical examinations”) with their frequency assessed on a 5-point Likert scale (from 1 – almost never to 5 – almost always). The internal consistency index value for the health behaviors scale was *α* = 0.821.

### Analytical Strategy

Path analysis was used to model the potential moderating role of education type, age, and gender in the relationship between the variables included in the model. The type of acquired education as representing groups primarily focused on either spirituality or health-related behaviors coded dichotomously: 0 = education focused on physical health and the human body as the reference group and 1 = education focused on the human mind and spirit. The unstandardized path coefficient of the type of education is thus interpreted as “to acquire education on psychosocial health and the human mind and spirit,” and its value represents mean differences between the two groups. Gender was coded similarly, i.e., 0 = men and 1 = women. Additionally, a linear regression analysis was conducted to further examine the noted relationships between both types of education.

## Results

**Figure 2:**
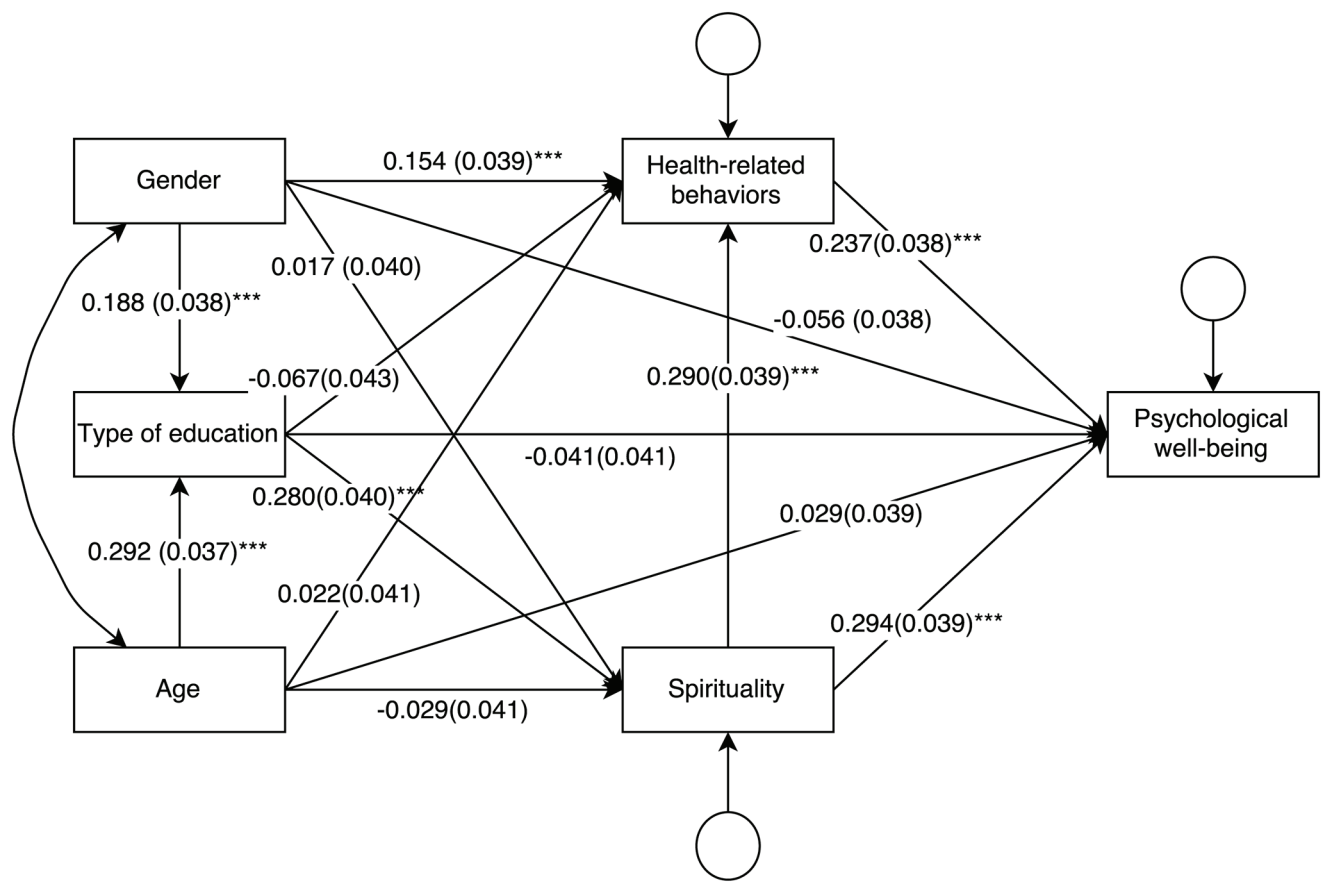
The tested model with standardized coefficients (standard errors in brackets).

The analysis was conducted using the Mplus 7 software package (Muthén and Muthén, 2012) and tested the model shown in Figure 1. Alternative models with different path directions acquired the same fit as the tested model (AIC = 5886.629, Sample-Size Adjusted BIC = 5901.195). The tested model is non-recursive and just-identified, thus, no statistic for absolute model fit can be assessed since they are uninformative. The model coefficients were calculated applying estimation based on the maximum likelihood. Standardized coefficients (StdYX) are presented in Figure 2. Table 2 contains both unstandardized and standardized coefficients. The paths were tested using the standard Sobel test (Sobel, 1982), yet due to some arguments of untrustworthiness (Hayes and Scharkow, 2013), 95% confidence intervals from a percentile-based bootstrap with 10,000 draws were also used.

**Table 2:** Unstandardized and standardized estimates, standard errors, *p*, and bootstrap C.I. for paths in the model (*n* = 595).

Path		Unst. est.	*SE*	St. est.	*p*	lower 2.5% C.I.	upper 2.5% C.I.
**Health-related behaviors → Psychological well-being**			
Direct		0.006	0.001	0.237	<0.001	0.004	0.008
Total indirect		-	-	-	-	-	-
Total		0.006	0.001	0.237	<0.001	0.004	0.008
**Spirituality → Psychological well-being**			
Direct		0.163	0.024	0.294	<0.001	0.115	0.211
Indirect through	Health-related behaviors	0.038	0.009	0.069	<0.001	−0.024	0.001
Total indirect		0.038	0.024	0.043	<0.001	0.022	0.057
Total		0.201	0.009	0.363	<0.001	0.156	0.247
**Type of studies → Psychological well-being**			
Direct		−0.026	0.026	−0.041	0.305	−0.076	0.024
Indirect through	Health-related behaviors	−0.010	0.007	−0.016	0.117	−0.024	0.001
Indirect through	Spirituality	0.053	0.011	0.082	<0.001	0.033	0.076
Indirect through	Spirituality						
	Health-related behaviors	0.012	0.003	0.019	<0.001	0.007	0.020
Total indirect		0.055	0.013	0.052	<0.001	0.029	0.082
Total		0.029	0.027	0.044	0.294	−0.025	0.083
**Gender → Psychological well-being**			
Direct		−0.038	0.026	−0.056	0.150	−0.091	0.013
Indirect through	Type of studies	−0.005	0.005	−0.008	0.322	−0.016	0.005
Indirect through	Health-related behaviors	0.025	0.008	0.037	0.002	0.011	0.041
Indirect through	Spirituality	0.003	0.008	0.005	0.699	−0.014	0.020
Indirect through	Type of studies						
	Health-related behaviors	−0.002	0.001	−0.003	0.144	−0.005	0.000
Indirect through	Health-related behaviors						
	Spirituality	0.001	0.002	0.001	0.707	−0.003	0.003
Indirect through	Spirituality						
	Type of studies	0.010	0.003	0.015	0.001	0.005	0.017
Indirect through	Health-related behaviors						
	Spirituality						
	Type of studies	0.002	0.001	0.004	0.004	0.001	0.004
Total indirect		0.034	0.014	0.016	0.015	0.008	0.062
Total		−0.004	0.028	−0.005	0.897	−0.060	0.050
**Age → Psychological well-being**			
Direct		0.005	0.006	0.029	0.437	−0.007	0.017
Indirect through	Type of studies	−0.002	0.002	−0.012	0.310	−0.006	0.002
Indirect through	Health-related behaviors	0.001	0.002	0.005	0.587	−0.002	0.004
Indirect through	Spirituality	−0.001	0.002	−0.009	0.511	−0.006	0.003
Indirect through	Type of studies						
	Health-related behaviors	−0.001	0.000	−0.005	0.123	−0.002	0.000
Indirect through	Health-related behaviors						
	Spirituality	0.000	0.000	−0.002	0.517	−0.001	0.001
Indirect through	Spirituality						
	Type of studies	0.004	0.001	0.024	<0.001	0.002	0.006
Indirect through	Health-related behaviors						
	Spirituality						
	Type of studies	0.001	0.000	0.006	0.001	0.000	0.002
Total indirect		0.001	0.003	−0.028	0.723	−0.005	0.008
Total		0.006	0.006	0.037	0.334	−0.006	0.018
**Type of studies → Health-related behaviors**			
Direct		−1.681	1.000	−0.067	0.093	−3.668	0.237
Indirect through	Spirituality	2.030	0.449	0.081	<0.001	1.217	2.959
Total indirect		2.030	0.449	0.053	<0.001	−1.669	2.342
Total		0.349	1.020	0.014	0.732	1.217	2.959
**Spirituality → Health-related behaviors**			
Direct		6.261	0.991	0.290	<0.001	4.334	8.204
Total indirect		-	-	-	-	-	-
Total		6.261	0.991	0.290	<0.001	4.334	8.204	(Continued)
**Gender → Health-related behaviors**			
Direct		4.043	1.038	0.154	<0.001	1.932	6.077
Indirect through	Type of studies	−0.332	0.213	−0.013	0.119	−0.788	0.048
Indirect through	Spirituality	0.126	0.328	0.005	0.701	−0.526	0.780
Indirect through	Spirituality						
	Type of studies	0.401	0.123	0.015	0.001	0.195	0.669
Total indirect		0.195	0.385	−0.017	0.612	−0.552	0.969
Total		4.238	1.066	0.162	<0.001	2.119	6.311
**Age → Health-related behaviors**			
Direct		0.137	0.246	0.022	0.578	−0.344	0.622
Indirect through	Type of studies	−0.123	0.075	−0.020	0.100	−0.274	0.018
Indirect through	Spirituality	−0.053	0.081	−0.008	0.510	−0.213	0.105
Indirect through	Type of studies						
	Spirituality	0.149	0.038	0.024	<0.001	0.084	0.232
Total indirect		0.109	0.255	−0.033	0.668	−0.387	0.615
Total		−0.027	0.109	0.017	0.800	−0.239	0.187
**Type of studies → Spirituality**			
Direct		0.324	0.048	0.280	<0.001	0.230	0.417
Total indirect		-	-	-	-	-	-
Total		0.324	0.048	0.280	<0.001	0.230	0.417
**Gender → Spirituality**			
Direct		0.020	0.052	0.017	0.697	−0.081	0.122
Indirect through	Type of studies	0.064	0.016	0.053	<0.001	0.035	0.098
Total indirect		0.084	0.053	0.031	0.113	−0.021	0.189
Total		0.064	0.016	0.069	<0.001	0.035	0.098
**Age → Spirituality**			
Direct		−0.008	0.013	−0.029	0.507	−0.033	0.016
Indirect through	Type of studies	0.024	0.004	0.082	<0.001	0.015	0.033
Total indirect		0.015	0.013	0.057	0.238	−0.010	0.041
Total		0.024	0.004	0.053	<0.001	0.015	0.033
**Gender → Type of studies**			
Direct		0.198	0.041	0.188	<0.001	0.112	0.265
Total indirect		-	-	-	-	-	-
Total		0.198	0.041	0.188	<0.001	0.112	0.265
**Age → Type of studies**			
Direct		0.073	0.009	0.292	<0.001	0.223	0.361
Total indirect		-	-	-	-	-	-
Total		0.073	0.009	0.292	<0.001	0.223	0.361

Education (0 = on the human’s body and 1 = on the human’s mind and spirit) and gender (0 = males and 1 = females).

The results indicated that both spirituality and health-related behaviors were directly related with psychological well-being (*p* < 0.001). Spirituality showed a positive relationship with health-related behaviors (*p* < 0.001). An indirect path of spirituality on psychological well-being through health-related behaviors was also distinguished (*p* < 0.001). The indirect relationship quantified the changes in well-being which are predicted by health-related behaviors associated with spirituality aside from the direct relationship (Alwin and Hauser, 1975). The structure of the model was theoretically well-established and the relationships had moderate sizes as represented by standardized coefficients, which suggested that they are justified and might be replicated in further research.

The direct relationship between the type of acquired education and psychological well-being was not significant (*M*_0_ = 4.52, *SD*_0_ = 0.31; *M*_1_ = 4.55, *SD*_1_ = 0.33; *p* = 0.305) as well as the relationship between education type and health-related behavior (*M*_0_ = 77.18, *SD*_0_ = 12.11; *M*_1_ = 78.35, *SD*_1_ = 12.90; *p* = 0.093), which demonstrated that both variables did not differ significantly between groups. The relationship between the type of education and spirituality was stronger in the human mind and spirit group (*M*_0_ = 3.41, *SD*_0_ = 0.55; *M*_1_ = 3.73, *SD*_1_ = 0.56; *p* < 0.001). Also an indirect relationship of type of education and health-related behavior through spirituality was observed (*p* < 0.001), although it was rather weak.

Although no direct relationship between psychological well-being and type of education was found, indirect relationships were note with spirituality (*p* < 0.001) and both spirituality and health-related behavior (*p* < 0.001), but not with health-related behavior alone. Although these relationships are not direct, the results suggest that acquiring education on psychosocial health and the human mind and spirit might be associated with a stronger relationship of spirituality and health-related behaviors with psychological well-being.

Gender and age were control variables in the model since both are known to affect the type of education. More women attended studies focused on education about the human mind and spirit (*n*_f_ = 221) than men (*n*_m_ = 79), whereas the gender ratio in physical health and the human body group of students was more balanced (*n*_f_ = 166 vs. *n*_m_ = 129). This led to a number of relationships between gender and the type of university studies (*p* < 0.001). In consequence, some indirect relationships between gender and the type of studies were significant, whereas direct relationships were not. Age was slightly higher in the human mind and spirit group. The difference was significant (*M*_0_ = 21.15, *SD*_0_ = 1.56; *M*_1_ = 22.30, *SD*_1_ = 2.20; *p* < 0.001), thus, some indirect relationships with age were significant with the type of acquired university education.

Although path models assume causal inference, we should bear in mind that all causally related factors that were excluded from the model are by definition represented in the form of error terms (Pearl, 2012). The variance of psychological well-being explained by this model was *R* ^2^ = 0.175, which means that a large portion of it is accounted for in sources other than variables contained in the model.

**Figure 3:**
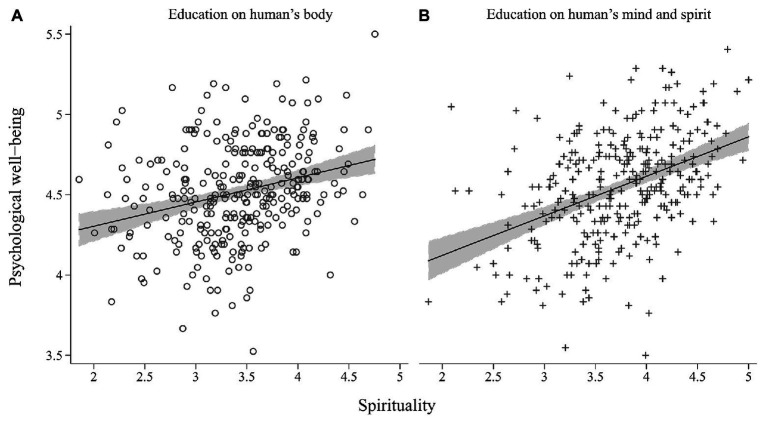
Correlation between spirituality and psychological well-being in group focused on human body (**A**; *r* = 0.273, *n*_0_ = 295) and human mind and spirit (**B**; *r* = 0.414, *n*_1_ = 300).

To gain a better insight into the role of education in the relationship of health-related behaviors, spirituality, and psychological well-being, a linear regression analysis with moderation terms was conducted (Table 3). The constant value (*b*_0_) represents the intercept of a group whose education is focused on the human body, whereas the predictor type of education corresponds to the difference between the group means. The intercept of psychological well-being was significantly lower (*b* = −0.495; *p* = 0.010) in the human mind and spirit group. The coefficient of health-related behavior in the group educated in physical health and the human body was significant (health-related behavior, *b* = 0.005; *p* = 0.001), and the human mind and spirit group did not differ from it significantly (Health-related behavior ^*^ Education = 1, *b* = 0.002; *p* = 0.455). The relationship between spirituality and psychological well-being was significant as well (Spirituality, *b* = 0.113; *p* = 0.001) but the slope was steeper in the human mind and spirit group (Spirituality ^*^ Education = 1, *b* = 0.098; *p* = 0.029; Figure 3). It can be thus concluded that spirituality has a stronger relationship with psychological well-being in university students whose curricula focus on the psychosocial dimension of health and the human mind and spirit.

**Table 3:** Results of the regression model predicting psychological well-being depending on health-related behavior, spirituality, and the type of education (*n* = 595).

Variable	*b*	*SE*	*β*	*p*	lower 2.5% C.I.	upper 2.5% C.I.
Education	−0.495	0.191	−0.771	0.010	−0.870	−0.121
Health-related behavior	0.005	0.001	0.201	0.001	0.002	0.008
Health-related behavior * Education	0.002	0.002	0.188	0.455	−0.002	0.005
Spirituality	0.113	0.033	0.203	0.001	0.048	0.177
Spirituality * Education	0.098	0.045	0.585	0.029	0.010	0.187
constant	3.733	0.132	-	<0.001	3.474	3.992

*R* ^2^ = 0.18; *F*(5, 589) = 25.96, *p* < 0.001; education (0 = on the human’s body and 1 = on the human’s mind and spirit).

## Discussion

The study revealed significant relationships between spirituality, health-related behaviors, and psychological well-being, in terms of the type of acquired education. The results indicate that both spirituality and health-related behaviors were associated with psychological well-being. The relationship between spirituality and psychological well-being was stronger in the human mind and spirit group of students. As longitudinal studies among adolescents by Kor et al. (2019) show spirituality is stable over time and contribute to better subjective well-being. It may also be considered to be a fundamental character strength and a crucial factor of positive development. Thus, spirituality may as well strengthen psychological well-being. Moreover, Giannone and Kaplin (2020) confirm that existential thinking and the production of meaning may be related to mental health. In general, spiritual intervention programs also contribute to mental health and well-being (Sanyal et al., 2020). Moreover, spirituality showed a similar relationship with health-related behaviors and was indirectly associated with psychological well-being through health-related behaviors. In other words, it seems that spirituality is not only directly associated with psychological well-being, but also might be moderated by health-related behavior. This is consistent with existing research (Jesse and Reed, 2004; Park et al., 2009; Unterrainer et al., 2014) and is an indication that spirituality is, in fact, a determinant of psychological well-being prior to health-related behavior. Despite this, a cross-sectional study cannot verify this claim directly.

The type of acquired education was related only to spirituality, but not to health-related behavior or psychological well-being. The relationship was stronger in the human mind and spirit group. The type of education served as a criterion of division of students into classes based on different approaches to physical health and the human body or psychosocial health and the human mind and spirit, which in turn were expected to display a discrepancy in spirituality and health-related behavior. The relationship between the type of education and psychological well-being was expected to be non-significant, as there were no assumptions of differences in the level of well-being between those groups.

The fact that the type of education was not associated with health-related behavior was more intriguing. Only an indirect relationship between those variables through spirituality was found, but it had a small size and was probably spurious. This shows that concentrating either on physical health and the human body or on psychosocial health and the human mind and spirit may not be directly related to one’s healthy habits. There are probably other factors affecting this relationship such as education or culture in which a young person grows up.

What differentiates the two groups is how they address their spirituality. It seems that, in the human mind and spirit group, spirituality plays a greater role in influencing psychological well-being. It can be assumed that the choice of university studies is determined by a specific attitude toward spirituality and personal development. Students of the humanities and social studies should be interested in human psychological development; thus, they are prone to have an interest in spirituality and internal development. Such study programs are adequately fitted to the above interests. This is a presumption which would be worth testing in further research.

In the present study, the students’ age did not reveal any relationship to either spirituality, health-related behavior, or well-being. However, many investigations do indicate medium to high correlations between age and spirituality (Alexander et al., 1990; Zimmer et al., 2016). This might be due to the small age difference between the subjects (students between 18 and 30 years of age). It is possible that with the simultaneous examinations of adolescents, students, and middle-aged people these differences would be significant.

The research findings may be a valid contribution to the discussion on the development of study programs focused on improving and maintaining various dimensions of human health and well-being. Modern university study programs often lack deep philosophical content, which should play a significant role in shaping the spirituality of young people. The commercialization of modern culture and marginalization of the humanistic education have removed the need to seek the meaning of life and reflect on the purpose of life. It seems that in a postmodern culture mostly focused on fulfilling the material needs of individuals, it is worth investing in the development of resources associated with spirituality. As demonstrated by Cotton et al. (2009), spiritual well-being is positively correlated with emotional and existential well-being, and it is also negatively correlated with symptoms of depression in adolescents. In contrast, Jafari et al. (2010) noted a significant relationship between spiritual well-being and mental health. Therefore, the results of the present study may find some practical application in the area of education.

Certain limitations of the study must be addressed. Firstly, the present study had a cross-sectional scope, and the subjects were not randomized between the groups. Thus, the results were not controlled for other inter-group variables. Further research is necessary, preferably using a longitudinal design allowing for comparisons before and after the choice of education type. Secondly, we used only self-report methods to measure all variables. As the survey was conducted among groups of young people who studied together for a number of years, the tendency toward social desirability might have biased participants’ answers. Thirdly, although we tried to diversify the study group by conducting studies in both state and private universities from different Polish cities, still the choice of particular majors and not involving others focused on the human body (e.g., medicine) or the human mind and spirit (e.g., religious studies) might have affected the results. We also did not explore the relatively larger number of students from other academic centers, and the study programs of the same majors may differ in part due to institutional autonomy. Fourthly, according to many researchers mature spirituality and religiosity are characteristic of people over 30 years of age (e.g., Fowler’s theory of stages of faith development; Fowler, 1981). To gain some more reliable knowledge about the relations between the studied constructs, it may be necessary to repeat the questionnaire survey in older groups. Fifthly, we did not consider such other determinants as attitudes toward lifestyle or cultural and socio-economic factors, which may affect the examined variables. Another limitation is that the tested model did not include separate subscales but rather general scores of each measure. The decision to use an elementary model was dictated by the lack of theoretical assumptions about the relationships between various measures to be tested. In fact, more complex associations may exist within different aspects of measured constructs. However, without theoretical assumptions, an exploratory approach might lead to spurious conclusions. Finally, the study results are limited to Poland only. It would be interesting to conduct research in more diverse environments.

Spirituality and health-related behaviors can play a significant role in defining psychological well-being. Personal focus on physical health and the human body or psychosocial health and the human mind and spirit, might also determine psychological well-being. However, these claims require more research, especially involving a comprehensive and analytical approach to various types of health-related behavior, different forms of spirituality, and detailed aspects of psychological well-being. Further research is also necessary to explore other determinants of the choice of university studies, e.g., specific attitudes toward health, spirituality, and personal development.

The findings of the study supplement the existing literature by indicting that multiple pro-health behaviors are positively related to psychological well-being. The study provides valuable information for faculty members responsible for curriculum development – not only in the context of higher education – but also for the enhancement of the contents of their educational programs with activities encouraging young people to lead a healthy lifestyle and build a healthy and resourceful society.

## Data Availability Statement

The raw data supporting the conclusions of this article will be made available by the authors, without undue reservation.

## Ethics Statement

Ethical review and approval was not required for the study on human participants in accordance with the local legislation and institutional requirements. The patients/participants provided their written informed consent to participate in this study.

## Author Contributions

PN and AB conceived, designed, and executed the study. MB analyzed the data. PN, AB, and MB prepared the manuscript. All authors contributed to the article and approved the submitted version.

### Conflict of Interest

The authors declare that the research was conducted in the absence of any commercial or financial relationships that could be construed as a potential conflict of interest.
